# Predictive significance of ^18^F-FDG PET/CT metabolic parameters for the expression level of HER2 in gastric cancer

**DOI:** 10.3389/fonc.2025.1580166

**Published:** 2025-04-10

**Authors:** Zhang Shilai, Mo Shaozhou, Wei Linlin, Chai Hua, Pu Weiwei, Liu Ziya, Qiu Wenming, Yang Zhi, Liao Hai, Xiao Guoyou

**Affiliations:** ^1^ Department of Nuclear Medicine, Guangxi Key Clinical Specialty (Department of Nuclear Medicine), Guangxi Medical University Cancer Hospital, Nanning, Guangxi, China; ^2^ Department of Nuclear Medicine, The People’s Hospital of Guangxi Zhuang Autonomous Region, Nanning, Guangxi, China

**Keywords:** 18 F-FDG PET/CT, metabolic parameters, gastric cancer, HER2, metabolic tumor volume (MTV), total lesion glycolysis (TLG)

## Abstract

**Objective:**

To investigate the predictive value of pertinent metabolic parameters of ^18^F-FDG PET/CT in relation to the expression level of human epidermal growth factor receptor 2 (HER2) in patients with gastric cancer.

**Materials and methods:**

The data was retrospectively acquired from 105 patients who had been pathologically diagnosed gastric cancer prior to treatment at our institution, including clinical data, laboratory test results, histological information, ^18^F-FDG PET/CT metabolic parameters (including maximum standardized uptake value (SUVmax), mean standardized uptake value (SUVmean), peak standardized uptake value (SUVpeak), SUVmax normalized by lean body mass (SULmax), SUVmean normalized by lean body mass (SULmean), SUVpeak normalized by lean body mass (SULpeak), metabolic tumor volume (MTV), and total lesion glycolysis (TLG)), and HER2 expression level, from January 2018 to December 2022. The correlation between ^18^F-FDG PET/CT metabolic parameters and HER2 expression level was examined, and the predictive value of these measures for HER2 expression level was investigated.

**Results:**

Among the 105 patients, 27 exhibited positive HER2 expression, while 78 demonstrated negative HER2 expression. Significant differences in MTV and TLG between patients exhibiting positive and negative HER2 expression (*P* < 0.05). The best cut-off values for MTV and TLG were 20.3 cm³ and 72.3 g, yielding accuracy rates of 90.2% and 89.0% for predicting positive HER2 expression, respectively. Our further grouped study shows that in the gastric adenocarcinoma and Lauren classification groups, MTV was significantly negatively correlated with HER2 positivity. Notably, in mixed tumors, the AUC value reached as high as 0.85.

**Conclusions:**

The negative correlations between MTV/TLG and HER2 status demonstrated that HER2-positive tumors are associated with reduced metabolic burden, providing imaging biomarkers for clinical prognostic assessment. Notably, subgroup analysis in gastric adenocarcinoma and Lauren classification subgroups revealed significant negative associations between MTV and HER2 positivity, highlighting MTV’s potential utility in predicting HER2 expression across histological subtypes of gastric cancer and supporting its role in precision oncology.

## 1 Introduction

Gastric cancer remains a global health concern, ranking among the most prevalent malignancies with high incidence and mortality rates (1, 2). It not only severely impacts patients’ physical health but also brings about psychological stress and financial burdens to their families. In the progression of gastric cancer, human epidermal growth factor receptor 2 (HER2) plays a pivotal role. HER2 overexpression is associated with increased tumor aggressiveness, metastasis, and a poor prognosis (3, 4). Consequently, HER2 has become a key target for targeted therapies in gastric cancer treatment. Thus, accurately evaluating HER2 expression is of great importance in clinical practice.

Current methods for assessing HER2 in gastric cancer mainly include immunohistochemistry (IHC), fluorescence *in situ* hybridization (FISH), and chromogenic *in situ* hybridization (CISH) (5). IHC, which measures HER2 protein expression, is widely used for its simplicity. However, it suffers from inter-observer variability and inconsistent criteria, leading to potential misclassification (6).FISH and CISH can accurately detect HER2 gene amplification but are invasive, requiring tissue samples from biopsies or surgeries. They are also time-consuming and demand specialized skills. Moreover, these methods have limitations in detecting low - level or heterogeneous HER2 expression, calling for a better alternative.

Positron emission tomography/computed tomography (PET/CT) has significantly advanced cancer diagnosis and treatment monitoring (7, 8). In gastric cancer, 18F-FDG PET/CT can non-invasively visualize tumor metabolic activity by measuring glucose uptake, facilitating early detection, accurate staging, and treatment response evaluation (9, 10).Despite its wide application, the relationship between 18F-FDG PET/CT metabolic parameters and HER2 expression in gastric cancer remains unclear. Only a few recent studies have explored this connection, leaving ample room for further research (11). This presents an opportunity to investigate 18F-FDG PET/CT as a non-invasive predictor of HER2 expression, potentially revolutionizing HER2 assessment. Metabolic parameters, including metabolic tumor volume (MTV) and total lesion glycolysis (TLG), exhibit significant dependency on threshold selection during PET/CT-based tumor delineation. Given the well-documented threshold sensitivity of MTV/TLG quantification (12, 13), our analytical pipeline utilized the 40% SUVmax threshold method, aligning with both published evidence demonstrating its superior clinico-pathological correlations and our institutional imaging protocol standardization requirements. This dual-alignment strategy ensures comparability with existing literature while maintaining clinical interpretability of results.

This study aims to comprehensively explore the relationship between ^18^F-FDG PET/CT metabolic parameters and HER2 expression in gastric cancer patients. We hypothesize that specific ^18^F-FDG PET/CT parameters can accurately predict HER2 expression. If our hypothesis holds true, this could establish a novel non-invasive method for predicting HER2 status in gastric cancer. It would enhance the accuracy of HER2 assessment, guide personalized treatment, and help identify patients who can benefit from HER2-targeted therapies, ultimately improving patient outcomes and quality of life.

## 2 Materials and methods

### 2.1 Research subjects and inclusion/exclusion criteria

This study included gastric cancer patients pathologically confirmed from January 2018 to December 2022. Finally, a total of 105 patients were included in our study. The collected data included: patient gender, age, height, weight, carcinoembryonic antigen (CEA), cancer antigen 125 (CA125), cancer antigen 199 (CA199), cytokeratin fragment 21 (CYFR-21), Squamous Cell Carcinoma (SCC), lesion site, pathological type, histological grade, histological classification, maximum thickness of the lesion, SUVmax, SUVmean, SUVpeak, SULmax, SULmean, SUVpeak, MTV, TLG, and HER2 expression status. Inclusion Criteria: (1) All patients were newly diagnosed and pathologically diagnosed with gastric cancer through surgical resection or gastroscopic biopsy. (2) PET/CT scan was performed before surgical resection or gastroscopic biopsy. (3) Before the PET/CT scan, the patients had not received any anti - tumor treatment. (4) The case and imaging data were complete without any missing information. (5) The tissue specimens obtained from surgical resection or gastroscopic biopsy were all eligible for HER2 detection, and the detection was carried out in our hospital. Exclusion Criteria: (1) Patients with unclear pathological diagnosis or incomplete clinical data. (2) Patients who had received surgery, radiotherapy, chemotherapy, or immunotherapy before the PET/CT scan. (3) Patients with a history of malignant tumors in other parts.(4) Patients with severe heart, liver, kidney function disorders and other systemic diseases. (5) Patients who did not undergo HER2 detection.

### 2.2 ^18^F - FDG PET/CT scan

The whole - body PET/CT image acquisition was executed using a GE Discovery 710 PET/CT (GE Healthcare, America). The imaging procedure commenced 60 minutes subsequent to the intravenous injection of 18F - FDG through the peripheral cubital veins. The administered activity was precisely calculated based on the patient’s body weight, with a dosage of 5.55 MBq/kg. Prior to the administration of ^18^F-FDG, the serum glucose levels were maintained below 150 mg/dL.All patients first underwent non - contrast CT scanning, which was then followed by PET scans spanning from the skull to the mid - thigh level. The scanning process involved a total of seven or eight bed positions, with each position being scanned for 2.0 minutes. Positron emission tomography images were meticulously scatter - corrected and reconstructed by means of an ordered - subsets expectation maximization iterative reconstruction algorithm, accompanied by a post - reconstruction Gaussian filter (3 mm full - width at half - maximum).For the 64 - detector - row helical CT scanner, the technical parameters were set to yield a section thickness of 3.27 mm, under low - dose CT conditions with 140 kV and 110 mA.

### 2.3 Image acquisition and image processing

An attending physician and a nuclear medicine physician with a professional title of deputy chief physician or higher independently interpret the images while being acquainted with the relevant clinical history of the patient and other imaging examination data. The Medcalc software was employed for the reading and analysis of images, with the region of interest (ROI) being delineated. The computer measured the metabolic parameters of the gastric target lesions automatically, including SUVmax, SUVmean, SUVpeak, SULmax, SULmean, SULpeak, MTV, and TLG, based on the threshold = SUVmax × 40%. The consistent analysis results of the two physicians were regarded as the definitive diagnosis. In cases where the diagnostic opinions of two physicians are inconsistent, a consensus conclusion must be achieved through discussion and negotiation. A senior physician may be consulted to render the final decision if required.

### 2.4 HER2 expression detection

Tumor tissues were acquired via needle biopsy or surgical procedures for subsequent pathological and immunohistochemical analysis. HER2 diagnostic scoring criteria (1) 0: There was no staining or ≤10% of invasive cancer cells exhibited incomplete and faint staining of cell membrane. (2) 1+: ≥10% of invasive cells showed incomplete and faint staining of cell membrane. (3) 2+: >10% of invasive cancer cells exhibited weak to moderate intensity staining with complete cell membrane or ≤10% of invasive cancer cells demonstrated strong and complete staining of cell membrane. (4) 3+: >10% of invasive cancer cells exhibited strong, complete and uniform staining of the cell membrane. Positive HER2 expression was defined as 3+. When the score was 2+, additional *in situ* hybridization testing or replacement specimen testing was necessary to ascertain HER2 positive expression. Scores of 0 and 1+ were classified as HER2 negative expression.

### 2.5 Statistical analysis

SPSS 25.0 (IBM, Chicago, IL, USA) was employed to conduct all statistical analysis. Continuous variables were expressed as mean ± standard deviation. Counting data were denoted by n. Various clinical data and imaging metabolic parameters were analyzed and compared between groups using the t-test and Wilcoxon test (Mann-Whitney U method). The prediction of HER2 expression was evaluated using ^18^F-FDG PET/CT-related metabolic parameters with the receiver operating characteristic (ROC) curve. The optimal cut-off value was determined by maximizing the Youden index (Youden’s J = sensitivity + specificity − 1) through ROC curve analysis. The area under the curve (AUC) was calculated to evaluate the discriminative power of the variable. Initially, univariate correlation analysis was conducted all variables, and subsequently, multivariate logistic regression analysis was conducted to investigate the effect of influencing factors, independent influencing factors, and the extent of their impact on HER2 expression. Statistically significant was defined as *P* < 0.05.

## 3 Results

### 3.1 Clinical and pathological characteristics of patients

**Figure 1:**
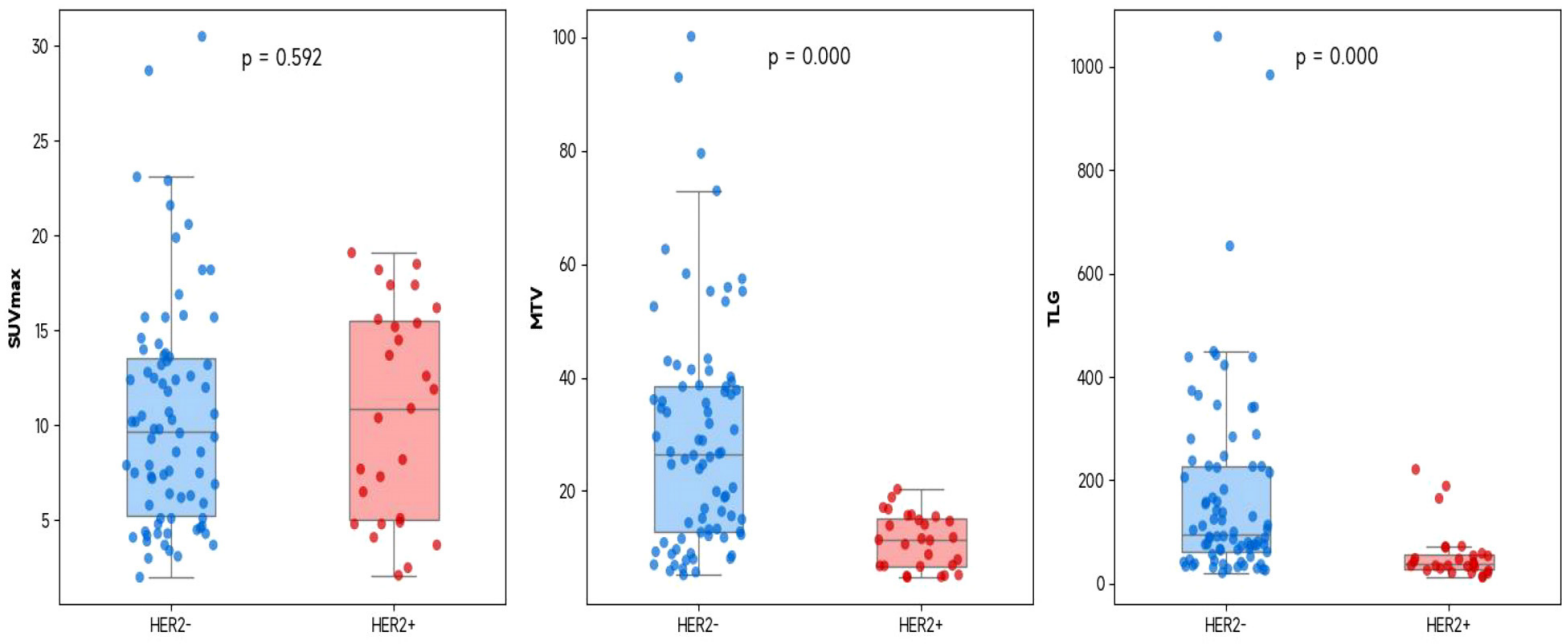
The correlation between the PET/CT metabolic parameters (SUVmax, MTV, and TLG) and HER2 expression in all gastric cancer patients (It is represented by a box plot superimposed with a jitter scatter plot, and the P value is obtained by using the Mann-Whitney U test).

Considering the insufficient sample size of uncommon gastric cancer subtypes (n=1 for squamous cell carcinoma and n=0 for adenosquamous carcinoma), which precluded meaningful statistical analysis, these rare variants were omitted from HER2 assessment to maintain analytical validity. The investigation was therefore restricted to gastric adenocarcinoma and signet-ring cell carcinoma cases, which constituted the majority of our cohort with adequate sample representation. The study comprised 105 patients, consisting of 59 males (56.1%) and 46 females (43.9%). The ratio of males to females was 1.28:1. The patients ranged in age from 33 to 83 years, with a mean age of 57 ± 11.82 years and a median age of 58 years. Histological grading revealed 5 well-differentiated tumor cases, 29 moderately-differentiated tumor cases, and 71 poorly-differentiated tumor cases, including 11 signet ring cell carcinoma cases. In the histological classification, there were 54 cases of the diffuse type, 31 cases of the intestinal type, and 20 cases of the mixed type. There were 27 cases with positive HER2 expression and 78 cases with negative HER2 expression. The HER2 expression positive rate was 25.7% (27/105), while the negative rate was 74.3% (78/105). In all 11 cases of signet ring cell cancer, HER2 expression was negative. Univariate analysis indicated that patient gender, age, CEA, CA125, CA199, CYFR-21, SCC, lesion location, pathological type, histological grading, histological classification, SUVmax, SUVmean, SUVpeak, SULmax, SULmean, and SUVpeak exhibited no statistically significant differences concerning HER2 expression levels (all *P* > 0.05). However, MTV and TLG were statistically different in relation to the HER2 expression levels,

The values of MTV and TLG in the HER2-positive group were significantly lower than those in the HER2-negative group (all *P* < 0.001) (**Tables 1**, **2**, **Figure 1**).

**Table 1:** The correlation between HER2 expression and clinicopathological characteristics of gastric cancer patients (n = 105) (categorical variables).

Clinicopathological characteristics	Number of examples	HER2 expression		*P*-value
		Positive (n=27)	Negative (n=78)	
Sex				0.523
male	59 (56.1%)	16 (15.1%)	43 (55.2%)	
female	46 (43.9%)	11 (10.4%)	35 (44.8%)	
CEA				0.127
Positive	40 (38.1%)	13 (12.3%)	27 (34.6%)	
Negatives	65 (91.9%)	14 (13.2%)	51 (65.4%)	
CA125				0.342
Positive	42 (40.0%)	12 (11.3%)	30 (38.4%)	
negatives	63 (60.0%)	15 (14.2%)	48 (61.6%)	
CA199				0.422
Positive	46 (43.9%)	13 (12.3%)	33 (42.3%)	
Negatives	59 (56.1%)	14 (13.2%)	45 (57.7%)	
CYFR-21				0.533
Positive	67 (63.8%)	18 (16.9%)	49 (62.8%)	
Negatives	38 (36.2%)	9 (8.5%)	29 (37.2%)	
SCC				0.324
Positive	4 (3.8%)	0 (0%)	4 (5.1%)	
Negatives	101 (96.2%)	27 (25.5%)	74 (94.8%)	
Lesion site				0.228
gastric sinus	40 (38.1%)	9 (8.5%)	31 (39.7%)	
pylorus	2 (1.9%)	2 (1.9%)	0 (0%)	
corpuscles	44 (41.9%)	11 (10.4%)	33 (42.3%)	
cardia	12 (11.4%)	3 (2.8%)	9 (11.5%)	
gastric fundus	7 (6.4%)	2 (1.9%)	5 (6.5%)	
Pathological type				0.083
Adenocarcinoma	94 (89.5%)	27 (25.5%)	67 (85.8%)	
Signet-ring cell carcinoma	11 (10.5%)	0 (0%)	11 (14.2%)	
Histological grade				0.611
Highly differentiated	5 (4.8%)	4 (3.8%)	1 (1.2%)	
Moderately differentiated	29 (27.6%)	10 (9.4%)	19 (24.3%)	
low polarization	71 (67.6%)	13 (12.3%)	58 (74.5%)	
Lauren classification				0.612
diffuse	54 (51.4%)	11 (10.3%)	43 (55.1%)	
enteric	31 (29.5%)	10 (9.4%)	21 (26.9%)	
hybrid	20 (19.1%)	6 (5.7%)	14 (18.0%)	

**Table 2:** The correlation between HER2 expression and clinical and PET/CT metabolic parameters of gastric cancer patients (n = 105) (continuous variables).

Considerations	Min-Max	Midpoint	Mean ± standard deviation	*P*-value
Age (years)	33-83	58	57 ± 11.82	0.360
Height (cm)	145-184	162	161.2 ± 7.92	0.800
Weight (kg)	30-90	55	56.67 ± 11.50	0.495
Maximum thickness of lesion (cm)	0.7-6.0	1.8	1.96 ± 0.91	0.404
SUVmax	2.0-30.5	10.0	10.54 ± 5.86	0.592
SUVmean	1.4-16.9	5.5	5.94 ± 3.27	0.783
SUVpeak	1.7-23.3	7.49	7.86 ± 4.34	0.812
SULmax	1.6-25.7	7.45	8.46 ± 4.77	0.766
SULmean	1.0-12.5	4.5	4.77 ± 2.66	0.692
SULpeak	1.2-17.7	5.75	6.31 ± 3.50	0.721
MTV (cm^3^)	4.8-100.1	17.0	24.93 ± 19.41	<0.001
TLG (g)	12.4-1058.8	76.1	144.36 ± 173.94	<0.001

### 3.2 The correlation between MTV and TLG of gastric cancer patients and HER2 expression

**Figure 2:**
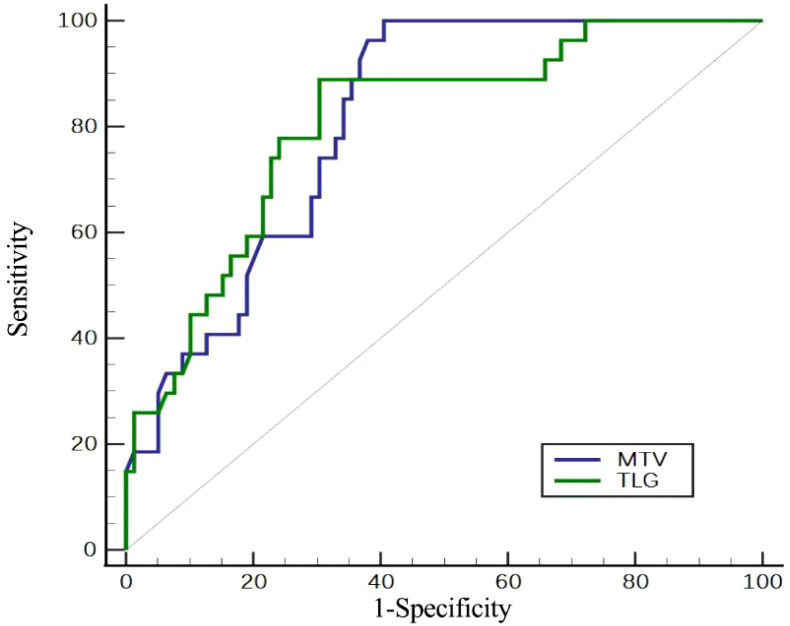
Assessment of the efficacy of PET/CT metabolic parameters in determining HER2 expression by ROC curve.

**Figure 3:**
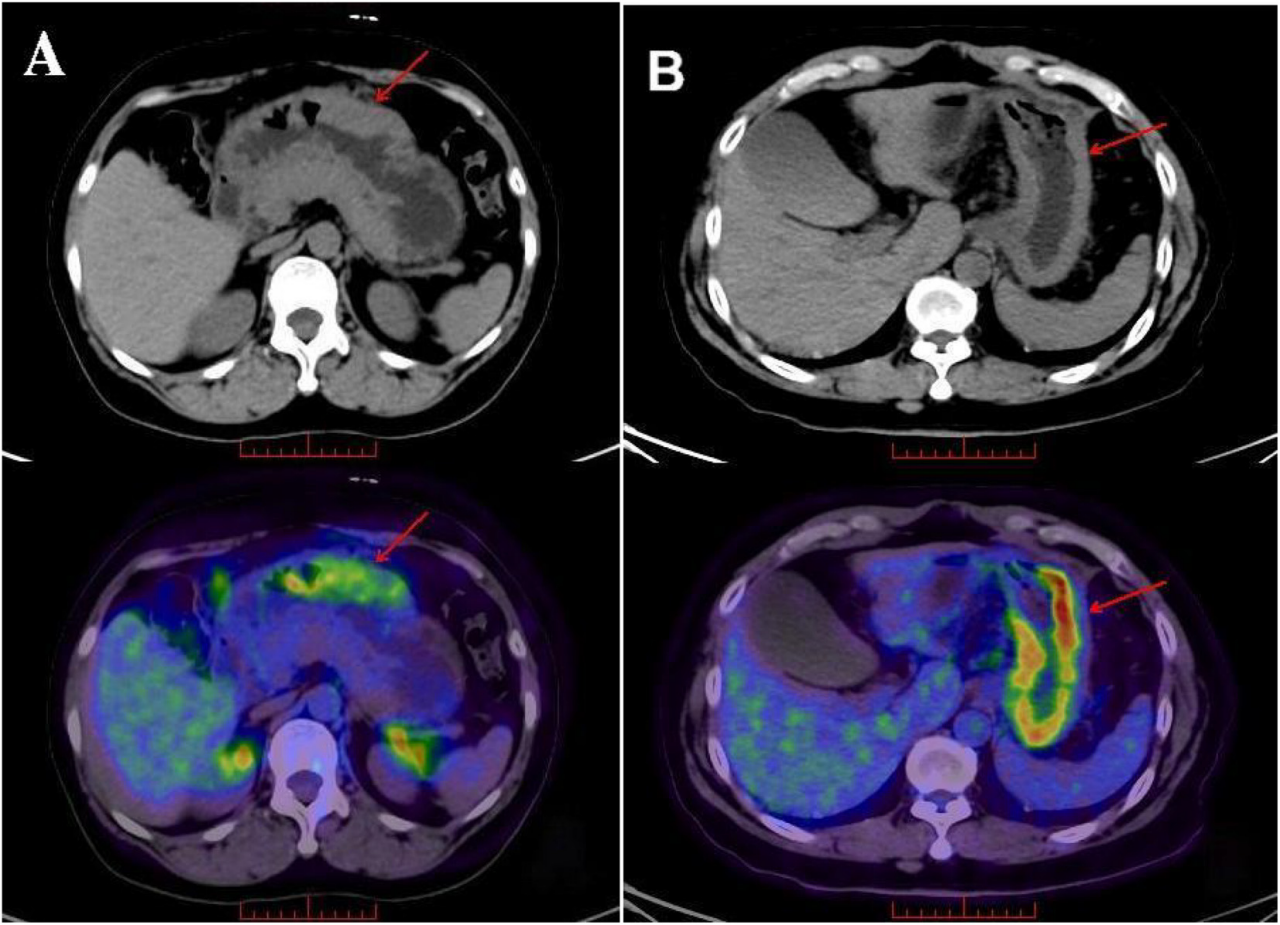
^18^F-FDG PET/CT images of HER2-positive **(A)** and HER2-negative **(B)** patients.

In order to predict HER2 positive expression, the ROC curve was used to determine the appropriate cut-off values for MTV and TLG of primary gastric cancer, which were found to be 20.3 cm³ and 72.3 g, respectively. HER2 positive expression was predicted with 90.2% and 89.0% accuracy, 96.3% and 88.9% sensitivity, 60.8% and 69.6% specificity, 45.8% and 50% positive predictive value, and 95.6% and 94.8% negative predictive value (**Table 3**, **Figure 2**). The positive expression rate of HER2 among patients with MTV ≤ 20.3 cm³ was 45.8% (27/59), significantly surpassing that in patients with MTV > 20.3 cm³ (0/46) (*P* < 0.001). In patients with TLG ≤ 72.3 g, the positive expression rate of HER2 was 50% (24/48), which was significantly higher than that of patients with TLG > 72.3 g (5.2%, 3/57) (*P* < 0.001) (**Table 4**, **Figure 3**).

**Table 3:** Optimal cut-off values for predicting positive HER2 expression by ROC curve.

Parameters	AUC (95% CI)	Optimal thresholds	Accuracy	Specificity	Sensitivity	*P*-value
MTV	0.81 (0.724-0.881)	≤20.3	90.2	60.8	96.3	<0.001
TLG	0.807 (0.719-0.877)	≤72.3	89.0	69.6	88.9	<0.001

**Table 4:** Analysis of the optimal cut-off values for HER2 expression (univariate analysis of the optimal cut-off values for HER2 expression).

Metabolic parameters	Number of examples	HER2 expression		*P*-value
		Positive (n=27)	Negative (n=78)	
MTV (cm^3^)				<0.001
≤20.3	59 (56.1%)	27 (25.5%)	32 (41.1%)	
>20.3	46 (43.9%)	0 (0%)	46 (58.9%)	
TLG (g)				<0.001
≤72.3	48 (45.7%)	24 (22.6%)	24 (30.8%)	
>72.3	57 (54.3%)	3 (2.9%)	54 (69.2%)	

In **Figure 3**, Figure A presents the ^18^F-FDG PET/CT image of a 56-year-old female gastric cancer patient with HER2-positive status (HER2 (3+, positive)). Thickening of the gastric wall along the greater curvature of the fundus-body of the stomach, accompanied by radioactive concentration, can be seen. The metabolic tumor volume (MTV) is 10.6 cm³ (less than 20.3 cm³), and the total lesion glycolysis (TLG) is 54.0 g (less than 72.3 g). Figure B shows the ^18^F-FDG PET/CT image of a 50-year-old male gastric cancer patient with HER2-negative status (HER2 (0, negative)). Diffuse thickening of the gastric wall in the fundus and body of the stomach, along with radioactive concentration, is observable. The MTV is 37.8 cm³ (greater than 20.3 cm³), and the TLG is 246.8 g (greater than 72.3 g).

### 3.3 Prognostic significance of MTV and TLG in predicting HER2 expression status across histological subtypes and molecular classifications of gastric carcinoma

In order to avoid introducing bias into the results, considering that the sample size of signet ring cell carcinoma is relatively small (n = 11) and all the HER2 expressions are negative, we conducted a multivariate analysis only on the patient samples of the gastric adenocarcinoma type and carried out cross-validation to assess the predictive efficacy. In addition, this study also analyzed and conducted cross-validation analysis on the predictive values of metabolic tumor volume (MTV) and total lesion glycolysis (TLG) in different subtypes of the Histological grade group and the Lauren classification group for the HER2 expression status. Due to the small sample size of the Highly differentiated group (n = 5), we combined it with the Moderately differentiated group (middle ground) and analyzed them as the same group.

Our results indicate that MTV significantly negatively predicts HER2 positivity in gastric adenocarcinoma (P=0.018), suggesting that an increase in tumor volume is associated with HER2 negativity. MTV may serve as a non-invasive predictor for HER2 negativity in adenocarcinomas. And the logistic regression model achieved a mean cross-validation accuracy of 85.7% (SD=3.2%) in predicting HER2 status in adenocarcinomas, suggesting robust performance. However, there was no significant association between TLG with positive HER2 expression (*P* = 0.805). Moreover, MTV demonstrated significant negative associations with HER2 positivity across all Lauren subtypes (Diffuse: β=-0.22, P=0.012; Enteric: β=-0.18, P=0.025; Hybrid: β=-0.30, P=0.001), suggesting its potential as a non-invasive biomarker for HER2-negative tumors. Lastly, In the Highly and Moderately differentiated group, MTV showed a trend toward negative association with HER2 positivity (β=-0.1345, P=0.0576), whereas TLG had no predictive value (P=0.5853).For Low polarization tumors, neither MTV nor TLG reached statistical significance (both P>0.25), likely due to limited sample size (n=23) (**Table 5**).

**Table 5:** Multivariable regression analyses were conducted to assess the independent predictive value of MTV and TLG for HER2 expression status across distinct histopathological subtypes and classifications of gastric carcinoma.

Classification and typing		Variable	β (95% CI)	P-value	AUC (95% CI)	Accuracy (Mean ± SD)
gastric adenocarcinoma		MTV	-0.15 (-0.28, -0.02)	0.018	0.78 (0.65, 0.89)	85.7% ± 3.2%
		TLG	-0.002(-0.02, 0.01)	0.805	0.52 (0.41, 0.64)	
Lauren classification	Diffuse	MTV	-0.22 (-0.39, -0.05)	0.012	0.76 (0.62, 0.90)	81.3% ± 4.7%
		TLG	0.001 (-0.02, 0.02)	0.92	0.51 (0.38, 0.64)	
	Enteric	MTV	-0.18 (-0.34, -0.02)	0.025	0.79 (0.67, 0.91)	87.1% ± 3.8%
		TLG	-0.003 (-0.03, 0.02)	0.75	0.55 (0.41, 0.69)	
	Hybrid	MTV	-0.30 (-0.47, -0.13)	0.001	0.85 (0.74, 0.96)	89.5% ± 2.9%
		TLG	0.005 (-0.01, 0.02)	0.4	0.58 (0.45, 0.71)	
Histological grade	High/Middle	MTV	-0.1345 (-0.27, 0.00)	0.0576	0.70 (0.58, 0.82)	75.6% ± 5.1%
		TLG	-0.0040 (-0.02, 0.01)	0.5853	0.52 (0.40, 0.64)	
	Low polarization	MTV	-0.1787 (-0.50, 0.14)	0.2597	0.85 (0.70, 1.00)	89.8% ± 4.2%
		TLG	-0.0205 (-0.09, 0.05)	0.5435	0.60 (0.42, 0.78)	

## 4 Discussion

In gastric cancer, HER2 expression level is closely linked to the selection of targeted therapy. Combining chemotherapy and anti-HER2 therapy is a standard treatment for patients with HER2-positive gastric cancer. In our study, the HER2 positive expression rate was 25.7%, aligning closely with the findings of prior research (14). Currently, significant discrepancies exist in the research findings about the forecasting of HER2 expression level in patients with locally advanced gastric cancer by serological methods. A statistically significant difference between HER2 expression level and CA199 level in patients diagnosed with gastric cancer has been observed in some studies. Zhou et al. (15) conducted a study on 256 gastric cancer patients, which did not reveal a statistically significant relationship between the level of CA199 and the level of HER2 expression. However, both HER2 positive expression level and CA199 level were identified as independent prognostic factors for gastric cancer patients. Our results demonstrated no significant association between tumor marker levels and HER2 expression level in gastric cancer patients. Consequently, it is uncertain if the levels of serological tumor markers can forecast HER2 expression in gastric cancer patients. This necessitates additional inquiry and validation via comprehensive, prospective, and multicenter clinical trials.

The interplay between HER2 overexpression and tumor metabolic reprogramming, particularly glycolytic activation, is increasingly recognized as a critical determinant of cancer progression and therapeutic resistance. HER2 amplification/overexpression activates downstream PI3K/AKT/mTOR signaling, which directly enhances glycolytic flux by upregulating glucose transporters (e.g., GLUT1) and glycolytic enzymes such as hexokinase 2 (HK2) and lactate dehydrogenase A (LDHA), with HIF1α and MYC acting as key downstream effectors (16, 17). These pathways not only fuel tumor growth but also contribute to therapeutic resistance and immune evasion. In HER2-positive gastric cancer, sustained activation of mTOR via AKT/ERK pathways promotes chromatin remodeling and YAP-dependent metabolic adaptation, driving resistance to trastuzumab (18). This pathway also correlates with elevated tumor glycolysis markers like MTV and TLG.

Many studies have sought to clarify the correlation between HER2 expression and FDG metabolism, but their findings exhibited considerable variability. Chen et al. (19) performed a retrospective investigation on 64 patients with gastric cancer before surgery. Signet ring cell carcinoma was precluded from the analysis, which revealed a significant association between the HER2 expression level and the SUVmax. In comparison to the positive group, the SUVmax in the HER2-negative group was significantly higher (8.619 ± 5.878 vs. 3.789 ± 2.613). The HER2 expression level was accurately determined with an SUVmax cut-off value of 6.2. However, studies conducted by Bai (20), Kim (21) and others have demonstrated that the SUVmax was significantly higher in the HER2-positive group than in the negative group. Furthermore, the results of our study were consistent with the work by Celli et al. (22), which found no statistically significant difference between the SUVmax and the HER2 expression level in the patients. The lack of statistical significance in SUVmax for evaluating HER2 expression in gastric cancer may be attributed to the complex regulation of metabolic activity in HER2-positive tumors by molecular subtypes, histological classifications, and co-expressed molecular pathways. For example: Heterogeneous activation states of HER2 signaling: Although HER2 overexpression typically promotes glycolysis via the PI3K/AKT/mTOR axis, compensatory metabolic changes may occur in tumors harboring PTEN loss or PIK3CA mutations, thereby attenuating direct correlations between SUVmax and HER2 status (23).SUVmax reflects only the peak metabolic intensity within a lesion, whereas HER2-induced metabolic reprogramming may manifest more robustly as changes in total metabolic tumor volume (MTV) or total lesion glycolysis (TLG) (24). For instance, studies in breast cancer have shown significant correlations between HER2 positivity and TLG80% (total glycolytic activity in 80% of the tumor volume) despite non-significant SUVmax differences (24). Furthermore the limited sample size of HER2-positive cases (n=27) in our study may have resulted in insufficient statistical power to detect subtle differences. The discrepancies between the previously mentioned study’s results and our study may be attributable to the following factors: (1) The inclusion and exclusion criteria for the study patients varied among different studies. Some studies included patients with locally advanced gastric cancer and advanced gastric cancer, whilst others exclusively involved patients with early-stage gastric cancer. Our study encompassed patients with early-stage gastric cancer, locally advanced gastric cancer and advanced gastric cancer. (2) The SUVmax is a semi-quantitative index, and many factors can influence the reliability of SUVmax. In the future, the relationship between HER2 and SUVmax needs to be further elucidated through standardized detection processes, an increase in the sample size, and the introduction of high-order radiomics features.

MTV and TLG, measured via PET/CT, are emerging as prognostic markers in HER2+ cancers, reflecting glycolytic activity driven by HER2/PI3K/AKT/mTOR signaling. Integrating metabolic biomarkers (e.g., MTV, TLG) and dual-targeting strategies offers promising avenues for improving outcomes in HER2-driven malignancies (18). MTV and TLG not only reflect the biological characteristics of tumor distribution in the body but also enhance the advantages of PET whole-body tumor monitoring (25). Theoretically, MTV and TLG, therefore, can more accurately represent the features of the entire tumor than SUVmax (26). Prior literature indicated (22, 27, 28) that MTV and TLG exhibited high sensitivity and specificity in forecasting the survival outcomes of cancer patients. However, the correlation between SUVmax and HER2 expression, as well as the prognosis of gastric cancer patients, remains unclear. Some studies have demonstrated (20, 29, 30) that, with the exception of signet ring cell gastric cancer, histological classification and TLG are independent factors for forecasting HER2 expression level. The positive expression of HER2 was elevated in patients with intestinal-type gastric cancer, and it was also higher in those with the primary gastric cancer lesion having a TLG ≤ 35.9 g. This validates the efficacy of ^18^F-FDG PET/CT in forecasting HER2 expression in gastric carcinoma. The results of our study revealed significant differences in MTV and TLG between patients exhibiting positive and negative HER2 expression (*P* < 0.01). The best cut-off values for MTV and TLG were 20.3 cm³ and 72.3 g, yielding accuracy rates of 90.2% and 89.0% for predicting positive HER2 expression, respectively. Our further grouped study shows that in the gastric adenocarcinoma and Lauren classification groups, MTV was significantly negatively correlated with HER2 positivity. Notably, in mixed tumors, the AUC value reached as high as 0.85. This result may be related to the biological characteristics of HER2 - positive tumors. HER2 - positive tumors typically exhibit higher proliferative activity and metabolic activity, yet they may be smaller in volume with clear boundaries. In contrast, HER2 - negative tumors may have a more invasive nature, leading to the spread of metabolically active regions and thus an increase in MTV (31). HER2 overexpression may promote glycolysis by activating pathways such as PI3K/AKT/mTOR. However, such tumors may be more dependent on specific metabolic patterns (such as high SUVmax rather than volume expansion), and an increase in MTV may reflect a non - HER2 - driven metabolic phenotype (32). The metabolic heterogeneity of mixed tumors may be more pronounced, and MTV can better capture their spatial heterogeneity, with the HER2 - negative sub - population potentially occupying a larger volume (33).But the small sample size in the Diffuse group (n=15) warrants cautious interpretation, and future studies with larger cohorts are needed to validate these findings. TLG did not show predictive efficacy in any of the groups. This may be because TLG integrates metabolic activity and volume, but the metabolic heterogeneity of HER2 - negative tumors may cause fluctuations in TLG values, reducing its discriminatory power (31). In the histological grade group, the marginal significance of MTV in High/Middle grade tumors suggests potential utility as a non-invasive biomarker, but further validation in larger cohorts is warranted. The high cross-validation accuracy in Low polarization group (89.8%) should be interpreted cautiously, as small sample sizes may inflate model performance metrics. The possible reason is that a small sample size may result in insufficient statistical power, and an expanded cohort is needed to verify the stability of the model.

Comparison and innovation of this study with existing research: In the imaging model, the cross - validation accuracy of the logistic regression model in some of our cohorts reached 85.7%, which is superior to the AUC of 0.7612 of the XGBoost model based on PET/CT in breast cancer (32). This suggests that the metabolic characteristics of gastric cancer may be more amenable to modeling, but this still requires large - sample and multi - center studies for confirmation. Regarding HER2 heterogeneity, similar studies have pointed out that the inconsistent expression of HER2 between the primary and metastatic sites (κ = - 0.056) may affect the generalization of the model, and multi - site sampling or dynamic monitoring is required (34). In terms of clinical translation potential, MTV, as a non - invasive biomarker, can assist endoscopic biopsy, especially in advanced patients where it is difficult to obtain tissue samples.

Although our study has provided valuable insights, several limitations are worth noting. Firstly, being retrospective, it lacks the randomization process in randomized controlled trials. This absence may introduce patient selection bias, thus distorting the relationship between PET/CT metabolic parameters and HER2 expression levels. Moreover, without a blinded methodology, observer bias may occur during the interpretation of PET/CT results, undermining the objectivity of data analysis. Additionally, selection bias regarding histological classifications exists, limiting the generalizability of the findings. Secondly, as a single - center clinical trial with only 105 patients, the small sample size fails to fully represent the heterogeneity of the gastric cancer patient population. This reduces the statistical power of the study, increasing the risk of overlooking true associations. In the future, our research will be improved in the following aspects. Multivariable adjustment: Incorporating clinical covariates (e.g., CRP levels for inflammation, tumor histology for heterogeneity) into regression models to isolate the independent effect of MTV/TLG. Radiomics-based refinement: Utilizing texture analysis of PET/CT images (e.g., entropy, homogeneity) to quantify tumor heterogeneity and integrate these features with MTV/TLG for improved predictive modeling. Dual-time-point imaging: Exploring delayed PET/CT scans to differentiate malignant uptake from inflammatory processes. Validation in controlled cohorts: Collaborating with multicenter cohorts that include patients with standardized pre-scan conditions (e.g., fasting glucose levels, no recent steroid use) to reduce variability.

## 5 Conclusion

The negative correlations between MTV/TLG and HER2 status demonstrated that HER2-positive tumors are associated with reduced metabolic burden, providing imaging biomarkers for clinical prognostic assessment. Notably, subgroup analysis in gastric adenocarcinoma and Lauren classification subgroups revealed significant negative associations between MTV and HER2 positivity, highlighting MTV’s potential utility in predicting HER2 expression across histological subtypes of gastric cancer and supporting its role in precision oncology.

## Data Availability

The datasets generated during and/or analyzed during the current study are available from the corresponding author on reasonable request.

## References

[1] Sung H Ferlay J Siegel RL Laversanne M Soerjomataram I Jemal A. Global cancer statistics 2020: GLOBOCAN estimates of incidence and mortality worldwide for 36 cancers in 185 countries. CA Cancer J Clin. (2021) 71:209–49. doi: 10.3322/caac.21660 33538338

[2] Smyth EC Nilsson M Grabsch HI Van Grieken NC Lordick F. Gastric cancer. Lancet. (2020) 396:635–48. doi: 10.1016/S0140-6736(20)31288-5 32861308

[3] Van Cutsem E Di Bartolomeo M Smyth E Chau I Park H Siena S. Trastuzumab deruxtecan in patients in the USA and Europe with HER2-positive advanced gastric or gastroesophageal junction cancer with disease progression on or after a trastuzumab-containing regimen (DESTINY-Gastric02): primary and updated analyses from a single-arm, phase 2 study”. Lancet Oncol. (2023) 24:744–56. doi: 10.1016/S1470-2045(23)00215-2 PMC11298287 37329891

[4] Ortiz-Martínez FP Perez-Balaguer AP Ciprián DB Andrés LM Ponce JM Adrover EPM. Association of increased osteopontin and splice variant-c mRNA expression with HER2 and triple-negative/basal-like breast carcinomas subtypes and recurrence. Hum Pathol. (2014) 45:504–12. doi: 10.1016/j.humpath.2013.10.015 24440093

[5] Kiyose S Igarashi H Nagura K Kamo T Kawane K Mori H. Chromogenic in *situ* hybridization (CISH) to detect HER2 gene amplification in breast and gastric cancer: Comparison with immunohistochemistry (IHC) and fluorescence in *situ* hybridization (FISH). Pathol Int. (2012) 62:728–34. doi: 10.1111/j.1440-1827.2012.02862.x 23121603

[6] Fusco N Rocco EG Del Conte C Pellegrini C Bulfamante G Di Nuovo F. HER2 in gastric cancer: a digital image analysis in pre-neoplastic, primary and metastatic lesions. Modern. Pathol. (2013) 26:816–24. doi: 10.1038/modpathol.2012.228 23348899

[7] Ruan D Zhao L Cai J Xu W Sun L Li J. Evaluation of FAPI PET imaging in gastric cancer: a systematic review and meta-analysis”. Theranostics. (2023) 13:4694–710. doi: 10.7150/thno.88335 PMC10465231 37649615

[8] Qin L Chen W Ye Y Yi H Pang W Long B. Prediction of HER2 expression in gastric adenocarcinoma based on preoperative noninvasive multimodal 18F-FDG PET/CT imaging”. Acad Radiol. (2024) 31:3200–11. doi: 10.1016/j.acra.2024.01.022 38302386

[9] Jiang Y Yuan Q Lv W Xi S Huang W Sun Z. Radiomic signature of 18F fluorodeoxyglucose PET/CT for prediction of gastric cancer survival and chemotherapeutic benefits. Theranostics. (2018) 8:5915–28. doi: 10.7150/thno.28018 PMC6299427 30613271

[10] Gertsen EC Brenkman HJF Van Hillegersberg R Van Sandick JW Van Berge Henegouwen MI Gisbertz SS. 18F-fludeoxyglucose–positron emission tomography/computed tomography and laparoscopy for staging of locally advanced gastric cancer: A multicenter prospective dutch cohort study (PLASTIC). JAMA Surg. (2021) 156:e215340–e215340. doi: 10.1001/jamasurg.2021.5340 34705049 PMC8552113

[11] Maron SB Chatila W Walch H Chou JF Ceglia N Ptashkin R. Determinants of survival with combined HER2 and PD-1 blockade in metastatic esophagogastric cancer. Clin Cancer Res. (2023) 29:3633–40. doi: 10.1158/1078-0432.CCR-22-3769 PMC10502449 37406106

[12] Jo HJ Kim SJ Kim IJ Kim S. Predictive value of volumetric parameters measured by F-18 FDG PET/CT for lymph node status in patients with surgically resected rectal cancer. Ann Nucl Med. (2014) 28:196–202. doi: 10.1007/s12149-014-0809-x 24532377

[13] Liao C Chen S Wu Y Chen WT Yen K Hsieh T. Correlations between 18F-FDG PET/CT parameters and pathological findings in patients with rectal cancer. Clin Nucl Med. (2014) 39:e40–5. doi: 10.1097/RLU.0b013e318292f0f6 24335567

[14] Jiang X Li T Wang J Zhang Z Chen X Zhang J. Noninvasive assessment of HER2 expression status in gastric cancer using (18)F-FDG positron emission tomography/computed tomography-based radiomics: A pilot study. Cancer Biother. Radiopharm. (2024) 39:169–77. doi: 10.1089/cbr.2023.0162 38193811

[15] Zhou H Dong A Xia H He G Cui J. Associations between CA19-9 and CA125 levels and human epidermal growth factor receptor 2 overexpression in patients with gastric cancer”. Oncol Lett. (2018) 16:1079–86. doi: 10.3892/ol.2018.8731 PMC6019918 29963185

[16] Kurebayashi J. Biological and clinical significance of HER2 overexpression in breast cancer. Breast Cancer (Tokyo Japan). (2001) 8:45–51. doi: 10.1007/BF02967477 11180765

[17] Fujimoto Y Morita TY Ohashi A Haeno H Hakozaki Y Fujii M. Combination treatment with a PI3K/Akt/mTOR pathway inhibitor overcomes resistance to anti-HER2 therapy in PIK3CA-mutant HER2-positive breast cancer cells. Sci Rep. (2020) 10:21762. doi: 10.1038/s41598-020-78646-y 33303839 PMC7729878

[18] Qiao J Feng M Zhou W Tan Y Yang S Liu Q. YAP inhibition overcomes adaptive resistance in HER2-positive gastric cancer treated with trastuzumab via the AKT/mTOR and ERK/mTOR axis. Gastric. Cancer. (2024) 27:785–801. doi: 10.1007/s10120-024-01508-3 38782859 PMC11193831

[19] Chen R Zhou X Liu J Huang G. Relationship between 18F-FDG PET/CT findings and HER2 expression in gastric cancer. J Nucl Med (1978). (2016) 57:1040–4. 10.2967/jnumed.115.171165 26966162

[20] Bai L Guo C Zhao Y Gao J Li M Shen C. SUVmax of 18F-FDG PET/CT correlates to expression of major chemotherapy-related tumor markers and serum tumor markers in gastric adenocarcinoma patients. Oncol Rep. (2017) 37:3433–40. doi: 10.3892/or.2017.5631 28498457

[21] Kim JS Young Park S. (18)F-FDG PET/CT of advanced gastric carcinoma and association of HER2 expression with standardized uptake value. Asia Ocean. J Nucl Med Biol. (2014) 2:12–8. PMC4937706 27408854

[22] Celli R Colunga M Patel N Djekidel M Jain D. Metabolic signature on 18F-FDG PET/CT, HER2 status, and survival in gastric adenocarcinomas. J Nucl Med Technol. (2016) 44:234–8. doi: 10.2967/jnmt.116.181479 27789750

[23] Li Y Dai L Wu X Zhao S Xu Y Jin X. Molecular characterization and classification of HER2-positive breast cancer inform tailored therapeutic strategies. Cancer Res (Chicago Ill.). (2024) 84:3669–83. doi: 10.1158/0008-5472.CAN-23-4066 39186675

[24] Marra A Chandarlapaty S Modi S. Management of patients with advanced-stage HER2-positive breast cancer: current evidence and future perspectives. Nat Rev Clin Oncol. (2024) 21:185–202. doi: 10.1038/s41571-023-00849-9 38191924 PMC12327481

[25] Antoch G Vogt FM Freudenberg LS Nazaradeh F Goehde SC Barkhausen J. Whole-body dual-modality PET/CT and whole-body MRI for tumor staging in oncology”. JAMA. (2003) 290:3199–206. doi: 10.1001/jama.290.24.3199 14693872

[26] Liu G Hu Y Cheng X Wang Y Gu Y Liu T. Volumetric parameters on (18)F-FDG PET/CT predict the survival of patients with gastric cancer associated with their expression status of c-MET. BMC Cancer. (2019) 19:790. doi: 10.1186/s12885-019-5935-3 31395059 PMC6686274

[27] Van De Wiele C Kruse V Smeets P Sathekge M Maes A. Predictive and prognostic value of metabolic tumour volume and total lesion glycolysis in solid tumours. Eur J Nucl Med Mol Imaging. (2013) 40:290–301. doi: 10.1007/s00259-012-2280-z 23151913

[28] Kim J Lim ST Na CJ Han Y Kim C Jeong H. Pretreatment F-18 FDG PET/CT parameters to evaluate progression-free survival in gastric cancer. Nucl Med Mol Imaging. (2014) 48:33–40. doi: 10.1007/s13139-013-0243-3 24900136 PMC4035159

[29] Park JS Lee N Beom SH Kim HS Lee C Rha SY. The prognostic value of volume-based parameters using (18)F-FDG PET/CT in gastric cancer according to HER2 status. Gastric. Cancer. (2018) 21:213–24. doi: 10.1007/s10120-017-0739-0 28643145

[30] Liu Q Li J Xin B Sun Y Wang X Song S. Preoperative 18F-FDG PET/CT radiomics analysis for predicting HER2 expression and prognosis in gastric cancer. Quant. Imaging Med Surg. (2023) 13:1537–49. doi: 10.21037/qims-22-148 PMC10006101 36915308

[31] Zhi H Xiang Y Chen C Zhang W Lin J Gao Z. Development and validation of a machine learning-based (18)F-fluorodeoxyglucose PET/CT radiomics signature for predicting gastric cancer survival. Cancer Imaging. (2024) 24:99. doi: 10.1186/s40644-024-00741-4 39080806 PMC11290137

[32] Chen Y Wang Z Yin G Sui C Liu Z Li X. Prediction of HER2 expression in breast cancer by combining PET/CT radiomic analysis and machine learning. Ann Nucl Med. (2022) 36:172–82. doi: 10.1007/s12149-021-01688-3 34716873

[33] Lu G Wang X Wang Y Cheng Z Zhou L. Value of CagA, HER2, ALDH1, and KiSS-1 in predicting metastasis and prognosis for gastric adenocarcinoma. Int J Clin Exp Pathol. (2018) 11:3628–37. PMC6962848 31949743

[34] Kim H Son S Woo CG Lee O Kim DH Yun HY. Discordance in HER2 status between primary gastric adenocarcinoma tumors and cells from the corresponding Malignant effusions. BMC Cancer. (2019) 19:834. doi: 10.1186/s12885-019-6035-0 31477048 PMC6721206

